# Including formal and informal caregivers in the development of Play Intervention for Dementia: a qualitative study

**DOI:** 10.1186/s12877-022-03232-y

**Published:** 2022-07-18

**Authors:** Bingyu Li, Rainbow Tin Hung Ho, Wing Yeung Vivian Leung, Ka Tat Tsang

**Affiliations:** 1 Department of Sociology, Shenzhen University, 3688 Nanhai Avenue, Shenzhen, Guangdong China; 2 Department of Social Work and Social Administration; Centre on Behavioral Health; Sau Po Centre on Ageing, The University of Hong Kong, Hong Kong, Hong Kong; 3 Factor-Inwentash Faculty of Social Work, University of Toronto, 246 Bloor Street W, Toronto, ON M5S 1V4 Canada

**Keywords:** Play intervention for dementia, Community-based participatory research, Caregivers of people with dementia, Contextualised dementia care

## Abstract

**Background:**

Play Intervention for Dementia is a practice initiative using play to help people living with dementia (PWD) experience engagements, autonomy, and cognitive stimulation. This program was developed under a participatory paradigm, with extensive contribution from formal and informal caregivers. This article describes how caregivers contributed to the practice principles, materials, and assessment during the development phase of Play Intervention for Dementia through community-based participatory research (CBPR).

**Methods:**

Three service supervisors, 16 formal caregivers and 14 informal caregivers from the community participated in this study. Based on CBPR, the study progressed in a reflexive, iterative and collaborative way. Data were collected from diverse sources, including practice journals, observation notes and reflexive focus group interviews. Two trained qualitative researchers conducted thematic analysis on the data collected, with focus on practical skills, outcomes, and caregivers’ general experience during the intervention.

**Results:**

The therapeutic and liberating power of play was thoroughly discussed by the caregivers. They considered play as an innovative way to understand, engage, and connect with the PWD. Also, improvement in energy level, motivation and communicative capacity was observed among the PWD. The researcher and caregivers collaboratively refined and designed the protocol of Play Intervention for Dementia, adding localized principles and games to the original design.

**Conclusion:**

Caregivers found play to be a meaningful way to engage with PWD, as it provided an equal platform for them. The intervention also enabled them to reflect upon ageing and disease at a deeper level. Caregivers have contributed significantly to the refinement and contextualisation of the intervention. The efficiency of the refined program should be further tested on a larger scale.

## Introduction

Dementia, as the greatest global challenge for health and social care in the twenty-first century [1], has long been a prevalent challenge for the elderly and caregivers. Caregivers have the most direct experience with the PWD and play significant roles in their lives. However, dementia interventions, mostly designed outside the practice contexts, generally overlook caregivers’ influence on intervention development, rendering their practice wisdom and needs unexplored. This ignorance hinders the knowledge transference and sustainability of interventions in real-life contexts [2], resulting in the prevalent knowledge-practice gap in the medical and social service systems [3]. As a social practice, research should not be separated from social reality, but rather engage with it in a constructive way. This study aims to transform the researcher-centred evaluation of dementia interventions by including voices of formal and informal caregivers in intervention development.

Caregivers’ working values construct the environment the PWD live in, and their personal relationship with the PWD can greatly influence the efficacy of dementia interventions. Physical and psychological burdens of dementia care are heavy, as dementia may raise caregivers’ death-related anxieties [4]. Therefore, interventions igniting positive interactions are needed by both the PWD and their caregivers.

As a meaningful and fundamental activity in human life, play is deemed an important activity for the PWD in the person-centred dementia care model [5]. It encourages the PWD to express themselves, enjoy happiness, build relationships and explore meanings in this new period of life [6]. It can also provide a realm for the PWD to more authentically share their experiences and construct counter-narratives against the dominant stigmatising discourses on dementia [7]. In addition, play is an inclusive concept that potentially integrates different evidence-based dementia care approaches [6]. Therefore, play may be a suitable form of dementia intervention, but few studies have explored this issue.

Several researchers have investigated the impacts of playing video games on the PWD [8, 9] and found that although it might increase positive mood and decrease loneliness, it had no significant effect on cognitive functioning or behavioral and psychological symptoms related to dementia [10]. Furthermore, it is difficult for PWD to play video games due to their physical and cognitive challenges [11]. As well, older adults generally prefer games that connect them with others and the society [12], while video games generally lack such feature.

Change in care services is individual, organisational, and socio-cultural. Many influential factors need to be considered for caregivers to truly adopt new skills or a different attitude in practice. Institutional support is necessary to enable caregivers to sustain the good practice, for example, by modifying work schedules, providing practice opportunities and changing treatment guideline policies [13]. Therefore, including caregivers’ voices in intervention development is crucial to the acceptance and adherence to the intervention, which are fundamental elements of successful implementation of a new intervention [14]. The purpose of this study was to investigate formal and informal caregivers’ perceptions and experiences of Play Intervention for Dementia and integrate their suggestions into future development of the intervention.

## Method

### Study design

This study adopted principles of community-based participatory research (CBPR) to integrate critical reflexivity, creativity, and coping flexibility of caregivers into intervention development. CBPR is a collaborative approach to research that equitably involves all partners in the research process and recognises their unique strengths. It acknowledges the interdependence of persons and their social environments and considers that knowledge is created in response to the diverse cultural, social and material needs of interest groups [15, 16]. CBPR begins with a research topic of importance to the community and combines knowledge and action for the improvement of community health and health equality [15, 17, 18]. Sustaining CBPR depends on trust-related mechanisms, continued commitment, mutual learning, and balanced power dynamics [17, 19]. In contrast to studies focused solely on outcomes, CBPR examines the context, group dynamics, intervention process, and outcomes [19].

The current study began with a local community’s need for an innovative dementia intervention, as many residents were living with dementia. However, direct implementation of knowledge produced in the Western context can be inappropriate or even dangerous to the local community [20, 21]. Therapeutic interventions need to be tailored according to the specific needs of the PWD and caregivers [22, 23]. In recent years, social work research has been increasingly committed to producing generalisable knowledge using scientific models, while in reality, such knowledge is seldom utilised in the practice field [24]. The failure to consider the active and agentive role of clients' and practitioners' voices may be one of the main reasons [25].

The study was conducted in two elderly service institutes within the same community in Hong Kong, including one nursing home and one day-care centre. The community suffered from a shortage of dementia care professionals, and therefore some services were provided by community volunteers, most of whom were also informal dementia caregivers.

### Intervention

The researchers first introduced to the community the basic principles of Play Intervention for Dementia, a practice initiative originally designed in Toronto [26], based on the Strategies and Skills Learning and Development System [27]. The program was designed with an open-ended protocol, encouraging professionals and practitioners to co-develop game plans, toys, and communication methods through action-reflection circles without pre-set rules. The key principle of the intervention was to facilitate entertaining activities, positive interactions, creative expressions, and autonomy through play among the PWD.

In each play session, participants played in groups of four to six for 60 to 75 min, and three to five groups could play simultaneously Games were rotated among the groups, with each game lasting eight to ten minutes to maximise stimulation. Games were specifically created to encourage the PWD to express themselves and break regular disciplines. For example, small balls used in typical occupational therapy were used to hit drums and produce music. To extend the concept of “play”, diverse tools, including toys, musical instruments, painting tools and other items, were also used. Different games encouraged the PWD to practise different capacities. For example, ball games involved many gross motor exercises; decoration games might improve fine motor skills and execution; shooting games required eye-hand coordination; card games involved calculation; pairing games involved short-term and long-term memories; and storytelling games might help to cultivate communicative capacity. Games varied in each session dependent on the functions and cultural backgrounds of participants. The pace of play was also adjusted according to the engagement and reactions of the PWD, and the play assistants would support them in realising their ideas and intentions without imposing rigid rules upon them.

The Strategies and Skills Learning and Development system conceptualises human life in six different yet interrelated domains: the body, motivation, emotion, cognition, behaviour, and environment [27]. All individuals are considered as active agents, and human behaviours are perceived as motivated and goal-directed [27]. Furthermore, human action is embodied and mediated by biological, cognitive, and emotional processes, and the ignorance of any of them can render a behaviour incomprehensible and thus unchangeable. Under this framework, caregivers are one of the agents of transformation in the practice context. The system values collaborative creation of new strategies and skills by client and practitioner to manage unfamiliar situations and address new challenges [27]. Therefore, changes start from eliciting and assessing the dynamic needs in the practice fields, followed by practice development tailored to these needs. In the process, openness and flexibility are promoted to create space for the elderly to express themselves. When the PWD can spontaneously play, the practitioners would support them without unnecessary rules and instructions.

### Participants

Three practice supervisors, 16 professional caregivers, and 14 informal caregivers participated in this study, forming a research alliance of diverse backgrounds and skills [28]. Purposive sampling was adopted for participant recruitment. The recruitment started with the researchers’ connection with the practice supervisor of the researched institutes. The supervisor introduced the program at the institutes and recruited interested professionals. Snowballing was then applied to recruit community-dwelling informal caregivers. Fourteen informal caregivers were selected based on their familiarity with the nursing home and dementia care.

Caregiver participants then invited the PWD they work with or live with to join the project [29]. The inclusion criteria of PWD were:

- Aged over 65;
- Diagnosed with one or more types of dementia;
- Did not recently suffer from severe illness;
- Could freely move their upper limbs.

Sixteen PWD joined the project, and no one dropped out. Written consent was collected from families of all PWD participants. Verbal consent from the participants was constantly sought by caregivers throughout the program. This study was reviewed and approved by the Human Research Ethics Committee of the University of Hong Kong (EA1701029). A detailed description of the intervention was published elsewhere [30]. The demographic information of the participants is listed in Table 1.

**Table 1:** Characteristics of caregiver participants and PWD participants (%)

Caregiver participants (*N* = 33)		PWD participants (*N* = 16)	
Female	31 (93.9%)	Female	12 (75%)
Age	44.9	Age	84.7
Occupation		Stage of Dementia^a^	
Service supervisors	3 (9.1%)	Severe	3 (18.8%)
Formal caregivers	16 (48.5%)	Moderate	10 (62.5%)
Informal caregivers	14 (42.4%)	Mild	3 (18.8%)

## Procedures

1. The researcher provided the participants with basic training on Play Intervention for Dementia.
2. The participants and the researcher collaboratively planned for and delivered weekly play intervention sessions for the PWD for eight weeks.
3. Both the researcher and the participants took notes of their observations, documented critical dialogues during the intervention implementation, and kept practice journals.
4. Focus group interviews were conducted with the participants after the eight-week program, where the participants collaboratively discussed their experiences in the program.
5. Interviews and documents were transcribed and analysed by two qualitative researchers.

### Data collection

CBPR generates diverse and discursive data, contributing to the development of both theory and practice. To capture a wide range of voices, various data sources were analysed, including focus group transcripts, practitioners’ practice journals and researchers’ observation notes.

Debriefing meeting minutes: each play intervention programme consisted of eight weekly sessions. After each session, the practitioners and volunteers gathered for a debriefing session in which they described their observations of the PWD, including their behaviours and changes during the session.

Focus groups: focus group interviews [31] were conducted with all participants at both sites after the program finished. Interviews were conducted to explore caregivers’ experiences with the program, the observed outcomes of the program, and most importantly, their suggestions for future development. All interviews were audiotaped and transcribed.

### Data analysis

The General Inductive Approach [32] was adopted to analyse the data, as it focuses on incoherent, small and conversationally situated narratives [33, 34]. All texts (focus group interview transcripts, meeting minutes and written testimonials) were analysed by two qualitative researchers using data-driven thematic analysis. The researchers followed the sequence of separate open coding, collaborative development of a codebook, and theme extraction [35]. Themes were finalised by all the authors through reflexive discussions. All the PWD, caregivers and practitioners are represented with pseudonyms in the following section. The validity of results was achieved through collective reflexivity and action implementation instead of generalizability [30].

## Results

Three main themes were generated from the analysis: ‘effects of play’, ‘practice principles of play’ and ‘feasibility of the program’. Each theme is further divided into different sub-themes.

### Theme 1: effects of play

#### Play boosts energy

The most apparent change identified was the improved energy level of the PWD. Caregivers reported that the PWD demonstrated higher energy during play sessions in comparison to other routine activities. One service supervisor said:

“The participants have also participated in other interventions, including cognitive stimulation groups and reality orientation groups, but the energy is different. They appear to be more excited, engaged more actively and demonstrated more capacities in the playgroups.” (Informant #1)

One informal caregiver believed that play provided the PWD with motivation and stimulation:

“It’s unusual to see them maintain such high energy for 75 minutes. Normally they make many excuses to resist physical and mental training, such as making bathroom calls or complaining about pain and hunger. But they are quite active during play…I notice that in daily exercises, the PWD lack motivation, while during play, they actively push themselves to their limits.” (Informant #21)

#### Play as catharsis

Emotional expression during play was also a well-discussed theme among the caregivers. Some volunteers and caregivers became more attentive to the PWD’s emotional needs and developed empathy towards them during the intervention:

“I didn’t know that they needed a way to release their emotions, be it anger, depression, or hopelessness. It made me realise the sufferings they have gone through. Maybe they came to nursing homes against their will; maybe they are bothered by how little their relatives come to visit them, or maybe they just can’t adjust themselves to their current state as they used to be highly successful people…” (Informant #4)

As the intervention progressed, some caregivers gained new understanding of the PWD. Noticing and understanding the emotions expressed by the PWD was the first step in person-centred care. In this way, certain behaviours can be further interpreted as the expression of needs instead of symptoms related to dementia:

“They do need to release their emotions. Some older women were very focused and ferocious when they threw the sandbags…and every time they threw a sandbag, you can see their mood lightened up a bit. They need to keep playing. They need to first let out the suppressed emotions, and then they will talk to you genuinely.” (Informant #12)

#### Play as a way to communicate

Some professional caregivers considered play as a new way of communication. Involving both verbal and non-verbal expressions, play enables caregivers to talk to the PWD in creative ways. This new form of communication provides both parties with stimulation. One care worker said:

“To be honest, as a full-time care worker, I spend more time with them (the PWD) than my family. I know exactly how they spend their time. What should I talk to them? But I hate to see them sitting there with nothing to do. The games gave them new stimuli. And I’m impressed that many of them told you new stories about themselves during play. It’s actually an interesting way to communicate with them.” (Informant #11)

#### Play helps reduce caregivers’ stress

Work-related stress is another critical issue faced by care workers. Intensive care duties as well as the anxiety associated with deterioration and death create both physical and mental burdens. Care workers need to relieve the stress and lighten their mood, and play provided them with such opportunity. One care worker said:

“Play reduces stress of not only the PWD but also us care workers. We all need to cope with our stress.” (Informant #9)

A practice supervisor shared similar observation:

“In the playgroup, everyone’s goal is pure and simple: let the group members play happily. It’s a powerful time. We see the potential of the PWD as well as our staff. Many of the caregivers are not full-time staff, but they are quite devoted and creative. You never know how others may react during play, and the happy moments become part of your memory.” (Informant #3)

### Theme 2: principles of play

Caregivers’ practice wisdom and hands-on experience greatly contributed to the principles of the intervention. The following principles were added to the intervention training materials after the study.

#### Play beyond games

The first and foremost principle summarised by the caregivers is that play is beyond games. Although games are widely used in different dementia interventions, the rigid rules, as well as the competitive nature, sometimes restrain the PWD from fully expressing themselves and fulfilling their potential. In addition, simply introducing games to the table rarely leads the PWD to the ‘play zone’. Therefore, play is beyond rules and rigid formats; instead, it is a fluid spirit. In one caregiver’s words,

“Play has to go beyond rules. Their lives are already strictly disciplined. They need to play freely and not care about any rule…I think play doesn’t necessarily mean games. Some elderly, for example, Elisa, are not interested in toys. But there’s one time, we brought in girl scout costumes, and she excitedly put them on, telling stories about being a girl scout when she was young. So, we began to role play following Elisa’s lead and had a lot of fun. I think play is an attitude. It’s about freedom and joy.” (Informant #8)

#### Build trust

Trust is another crucial element in play. Both formal and informal caregivers considered trust as the core of authentic play:

“Trust is important. They have to believe that they can tell you everything, and even if they irritate you from time to time, they can still fix it. With trust, they can play freely. It’s easier for us to talk to them when we can make fun of everything.” (Informant #2)

Besides personal relationship with the PWD, building a trusting and friendly group environment during the play sessions was also important:

“Group (cohesion) is the key. Many of them may feel embarrassed about playing games like little kids, but when they see others playing it, they feel safe and start to have fun. Annie (a PWD participant) once said during a game, ‘Oh we’re playing like children!’ and I asked her, ‘Aren’t we all children?’ ‘Yes, we are’, she answered with a smile. She became more active since that session.” (Informant #12)

#### Use pre-existing rapport

Existing relationships between the practitioners and the elderly were also valued. Most caregivers have extensive knowledge about the PWD they work with, and sometimes this knowledge can be translated into therapeutic moments in the play groups. One informal caregiver shared her observation of a magical change during practice:

“During self-introduction, Ben, an older man with severe dementia, repeatedly said ‘Obama,’ and some people didn’t understand. But Karen, a care worker who worked with him for long, explained to others that ‘Obama’ was a nickname he chose for himself after he had dementia. Later in the playgroups, Ben couldn’t understand the rules others suggested and responded with random words. Karen could understand what he meant and decode his expressions, thus connecting Ben with the rest of the group. The rapport between the caregivers and the PWD is thus a strong foundation for a successful playgroup.” (Informant #14)

#### Encourage PWD’s autonomy

As the intervention progressed, the participants began to notice deeper issues in dementia care. For example, some care workers started to reflect on the influence of disciplines on the PWD and realised that the PWD was still capable of doing many things when given autonomy:

“At the beginning, I was nervous to design so many games. Later, I found that play could spontaneously continue if I stopped worrying about the rules and followed the PWD’s ideas. They can’t have fun if we give them too many instructions. However, they were actually very creative when we encouraged them to play in whatever way they wanted. They can even be leaders and bring joy to us.” (Informant #17)

### Theme 3: feasibility of the program

The feasibility of the program was also discussed by the participants in terms of its strengths and limitations. In general, the program was well accepted by caregivers. Limitations related to human capital and resources, however, remain a concern.

#### Strength of this training model

Both formal and informal caregivers found CBPR to be an appropriate approach to intervention development as it provided them with adequate space for reflection and democratic knowledge generation. One service supervisor shared:

“It’s been a luxurious experience for me. We haven’t got a chance to engage with the PWD in such an intimate way and learn about their needs in such detail. The group reflections helped a lot. We started from knowing nothing about play intervention; after a series of reflections, I know so much more about the strengths, needs, and emotions of the PWD. It also helped me see the potential of my colleagues and myself.” (Informant #2)

#### Limitations

Human capital and resources, however, remain the biggest concern amongst the participants. A service manager said:

“Lack of support personnel can be a big problem. We’re lucky that we have enough people to run the program, but it’s indeed labour-intensive. I don’t know if it’ll still be this effective if only two to three staff run it.” (Informant #14)

Typically, each play group requires support from at least two caregivers (formal or informal). It may cause strain to service agencies if they do not have sufficient trained staff and volunteers. Future development of the intervention should consider ways to reduce the required number of supporting members or streamline the training process.

## Discussion

Adopting a participatory paradigm, this study generated valuable insights from caregivers regarding the positive impacts of Play Intervention for Dementia and the key principles of successful implementation. Feasibility was also explored in this specific practice context. This research identified the resources and support needed for the program to be consistently implemented.

### Acceptability of Play Intervention for Dementia

Caregivers in this project generally considered play as an important and meaningful activity for the PWD. Play was conceptualised as a realm for experience of interconnectivity, humour and contentment [6]. Play provided both the PWD and the caregivers with a stress-relieving space in which the PWD could freely engage in cathartic activities. These activities enabled caregivers to understand the PWD from new perspectives and adopt a more empathetic attitude toward them. Furthermore, play enabled the PWD to demonstrate higher level of motivation and energy than in their daily life.

### Feasibility of Play Intervention for Dementia

The feasibility of the program was discussed in terms of the resources required, difficulties of implementation and the self-efficacy of caregivers. Caregivers found reflexive discussion groups effective in improving their caregiving self-efficacy.

However, the lack of resources is a big concern to run this intervention. This intervention was deemed labour-intensive by many practitioners, because the PWD, especially those who were at moderate to severe stages of dementia, needed much facilitation and assistance during the play process. Two solutions may address these problems. First, the institution can turn each play session into a weekly practice development meeting where experienced caregivers can provide training to junior and informal caregivers. Second, informal caregivers and community-dwelling volunteers can be trained to support the intervention. Compared to other interventions, Play Intervention for Dementia is simpler and more comprehensible to people of different backgrounds. All informal caregivers in the present study gradually developed practice competence and self-efficacy during the eight-week program and considered the experience rewarding and meaningful.

#### Tailor-made games, person-centred care

Results show that play should be conceptualised as a guiding framework instead of specific and rigid game design. Interpersonal relationship, socio-cultural background, individual capacities, and other factors together influence the efficacy of the intervention. Therefore, caregivers’ attempts to create tailor-made games during the play sessions were successful in creating a safe and spontaneous play zone for the PWD.

For example, the cultural background of PWD has been found to impact the intervention. Most PWD experienced great pain in their childhood, including famine, wars, and poverty, and they generally considered play as a luxurious experience. Some toys rekindled their most precious memories from childhood and brought up touching stories to the playgroup. In addition, this generation of older people has witnessed a multitude of major events, and even with fragmented memories, they still have many invaluable narratives to share. Hence, culturally sensitive games were added to the playgroup based on the personal accounts of the PWD. The playgroup provides them with a safe space where they can spontaneously express themselves, communicate their perspective about the world to their caregivers, and most importantly, construct an authentic existence against the barriers from ageing and disease [7].

Previous studies found positive association between participation in leisure activities and cognitive functioning [36, 37]. Spontaneous play can serve as a realm for aesthetic experience, which is vital for preserving the selfhood of PWD, especially when the disease deteriorates [38]. The findings of the current study are in accordance with previous results on the effect of play, and they further demonstrate the possibility of developing play into a sustainable intervention in day-care centres and nursing homes.

#### Methodological contribution

By adopting a CBPR approach, the study generated experiential knowledge through collaborative exploration. Caregivers in this study offered their valuable insights on Play Intervention for Dementia based on their hands-on experiences and daily observations. Their accounts made visible the micro-processes in the playgroup. When developing interventions, researchers should include the voices of both formal and informal caregivers, so as to achieve higher efficacy, feasibility and sustainability. Furthermore, the experience of dementia is heterogeneous and context-specific; therefore, a reflexive, collaborative approach like CBPR can potentially contribute to the contextualisation of interventions.

### Limitations

There are several limitations in this study. First, PWD were not included in the focus group interviews due to the concern for their energy, health conditions and communicative capacity. The 75-min play session was a considerable physical challenge for many PWD, rendering post-session interviews too exhausting for them. Delayed interviews, on the other hand, might impose cognitive challenge on most PWD. To compensate for this limitation, detailed observations were conducted during the play session, followed by immediate post-session discussions focused on the PWD’s responses and reactions during the play session. Second, only two institutions were included in this study, and all participants were Chinese. The emphasis on contextualisation compromised ethnic diversity of this study. Third, the inclusion of both formal and informal caregivers enriched the research, but it also increased the challenges of coordination and progress management.

## Conclusion

This study explored the insights and experiences of caregivers in Play Intervention for Dementia and discovered that group play was a highly accepted type of intervention. Caregivers’ involvement has contextualised the intervention and further developed the practice principles by integrating local cultural elements and individual differences into game design. In addition, boosted energy, positive emotions, enhanced social interactions, and increased autonomy were identified as the positive outcomes of play. These insights contributed to both theoretical and practical developments of Play Intervention for Dementia. Furthermore, CBPR, as an iterative, reflexive, and democratic research methodology, were helpful in extracting the practice wisdom from caregivers, simultaneously improving their practical skills and self-efficacy. Future studies should further increase the involvement of caregivers in designing and implementing dementia interventions, in order to develop more sustainable dementia interventions that tailored to the emerging needs of PWD. 

## Data Availability

The datasets generated and/or analysed during the current study are not publicly available due to confidentiality concerns but are available from the corresponding author on reasonable request.

## Abbreviations

PWD: People living with dementia
CBPR: Community-based Participatory Research
